# The Role of Dicer Protein Partners in the Processing of MicroRNA Precursors

**DOI:** 10.1371/journal.pone.0028548

**Published:** 2011-12-06

**Authors:** Edyta Koscianska, Julia Starega-Roslan, Wlodzimierz J. Krzyzosiak

**Affiliations:** Laboratory of Cancer Genetics, Institute of Bioorganic Chemistry, Polish Academy of Sciences, Poznan, Poland; Beckman Research Institute of the City of Hope, United States of America

## Abstract

One of the cellular functions of the ribonuclease Dicer is to process microRNA precursors (pre-miRNAs) into mature microRNAs (miRNAs). Human Dicer performs this function in cooperation with its protein partners, AGO2, PACT and TRBP. The exact role of these accessory proteins in Dicer activity is still poorly understood. In this study, we used the northern blotting technique to investigate pre-miRNA cleavage efficiency and specificity after depletion of AGO2, PACT and TRBP by RNAi. The results showed that the inhibition of either Dicer protein partner substantially affected not only miRNA levels but also pre-miRNA levels, and it had a rather minor effect on the specificity of Dicer cleavage. The analysis of the Dicer cleavage products generated *in vitro* revealed the presence of a cleavage intermediate when pre-miRNA was processed by recombinant Dicer alone. This intermediate was not observed during pre-miRNA cleavage by endogenous Dicer. We demonstrate that AGO2, PACT and TRBP were required for the efficient functioning of Dicer in cells, and we suggest that one of the roles of these proteins is to assure better synchronization of cleavages triggered by two RNase III domains of Dicer.

## Introduction

The ribonuclease Dicer cleaves double-stranded RNA (dsRNA) into small interfering RNA (siRNA) and microRNA precursors (pre-miRNA) into microRNA (miRNA). Human Dicer belongs to the RNase III class and contains an N-terminal DExH-box RNA helicase-like domain, a domain originally termed the domain of unknown function (DUF283), a PAZ domain, two RNase III domains, and a double-stranded RNA-binding domain (dsRBD) [1]. The dsRNA processing center of Dicer is formed through intramolecular dimerization of two RNase III domains functioning together to cleave phosphodiester bonds on opposite strands of a dsRNA substrate [2]. The RNase IIIa domain cleaves the 3′-arm of pre-miRNA and the RNase IIIb domain cleaves its 5′-arm, and both domains must exhibit their activity to generate a miRNA-miRNA* duplex.

In human cells, Dicer does not act alone, but in cooperation with protein partners, such as members of the AGO family [3], [4], HIV-1 TAR RNA-binding protein (TRBP) [5], [6], a protein activator of PKR (PACT) [7], [8] and possibly other accessory proteins. The contribution of Dicer protein partners to either the efficiency or the specificity of miRNA biogenesis is important but is still unclear and poorly understood. It has been shown that Dicer, AGO2 and TRBP are necessary components in the formation of the RISC-loading complex (RLC) and the effective production of short RNAs [3]–[5], [9]. In one study, the depletion of TRBP affected pre-miRNA processing *in vitro*, but it had no effect on the accumulation of pre-miRNAs and mature miRNAs in cells [6]. In another study, the knockdown of TRBP in human cells was shown to result in lower levels of miRNAs, which suggested that the lack of TRBP decreased Dicer stability [5]. A similar destabilization of Dicer resulting from an impairment of TRBP was reported in the case of human carcinomas, in which frameshift mutations in the *TRBP* gene caused defects in the processing of miRNA precursors [10]. TRBP and PACT interact with each other and associate with Dicer to facilitate the cleavage of dsRNA [8]. The loss of PACT expression was also reported to influence the biogenesis of miRNAs [7].

Numerous approaches have been used to investigate how Dicer cleaves its pre-miRNA substrates, both *in vitro* and in cells. In the simplest system, synthetic miRNA precursors were cleaved by recombinant Dicer [2], [4], [11]–[14]. The results of these studies showed that the enzyme typically generates heterogeneous products from dsRNAs [15] and pre-miRNAs [2], [11], [13], [14]. The influence of the pre-miRNA structure on the length of the Dicer cleavage products has recently been demonstrated [14]. Two components of the RLC complex, Dicer and TRBP or Dicer and AGO2, were used to analyze cleavages in synthetic pre-miRNAs [5], [16]. Also, the pre-miRNA cleavages by Dicer present within the complete RLC reconstituted from recombinant Dicer, TRBP and AGO2 proteins were analyzed [4]. To demonstrate the synthetic pre-miRNA cleavage by endogenous complexes, Dicer activity from immunoprecipitates [3], [9], [17] and cellular extracts [11], [13], [18] was used, and synthetic precursors were injected into either the nucleus or the cytoplasm of *Xenopus* oocytes or early embryos [19].

Here, we studied the effect of Dicer protein partners on pre-miRNA cleavage efficiency and specificity. Close attention was paid to the length heterogeneity of generated miRNAs after depletion of AGO2, PACT, TRBP and Dicer by RNAi. We showed that the Dicer protein partners affect both miRNA and pre-miRNA levels and have a minor effect on the specificity of Dicer cleavage. We also transfected HeLa cells with synthetic pre-miRNAs and compared cleavage products with those generated *in vitro* by recombinant Dicer. The results revealed the appearance of a cleavage intermediate product when pre-miRNA was handled by Dicer alone and the absence of such an intermediate when exogenous pre-miRNA was processed by the endogenous Dicer complex. This suggests the role of Dicer's protein partners in the synchronization of cleavages triggered by RNase III domains.

## Results

**Figure 1:**
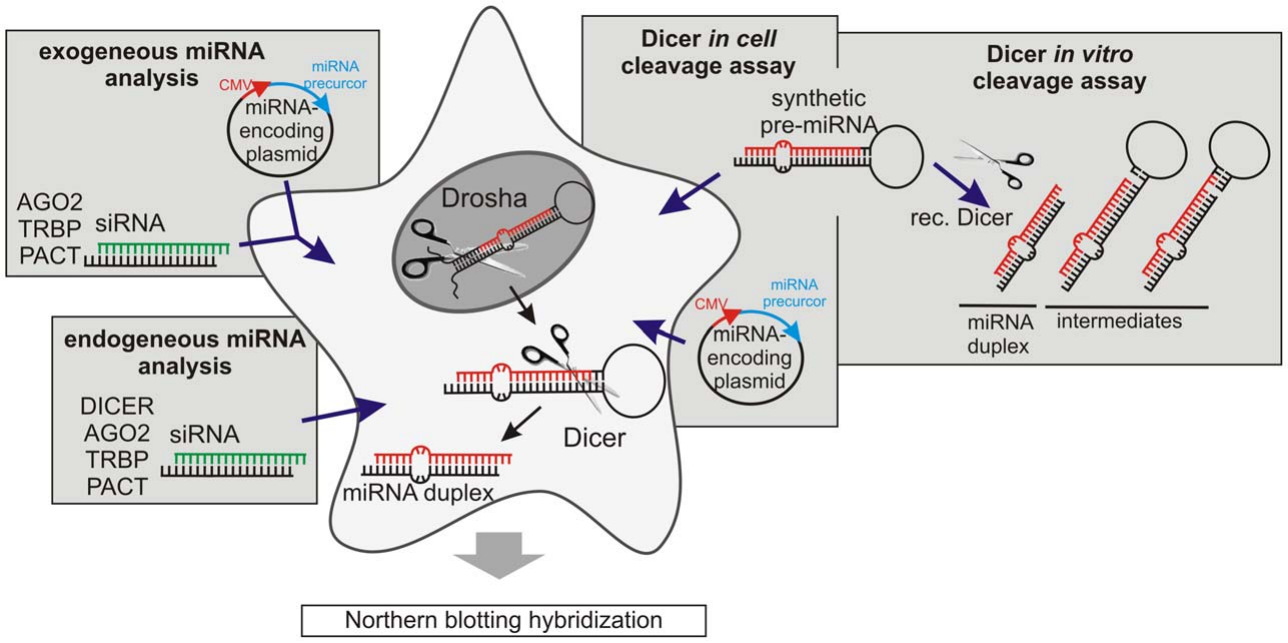
Approaches used to analyze the Dicer step of miRNA biogenesis in cells. Left: endogenous miRNAs expressed in HeLa cells were analyzed after depletion of AGO2, PACT, TRBP and Dicer with specific siRNAs, and exogenous miRNAs expressed from vector constructs were analyzed after depletion of AGO2, PACT and TRBP. Right: synthetic pre-miRNA precursors were transfected to cells or cleaved by recombinant Dicer, or pri-miRNA encoding vectors were transfected to cells. The results of all analyses were evaluated by northern blotting.

Dicer associates with several protein partners in cells, i.e., AGO2, PACT and TRBP. The role of these proteins in the processing of miRNA precursors has been investigated in several studies [3]–[5], [7]–[9], [16] but has not been satisfactorily resolved. To study the impact of Dicer protein partners on miRNA production in HeLa cells, we used two approaches. We evaluated the levels of both endogenous and exogenous miRNAs after the depletion of AGO2, PACT, TRBP and Dicer by RNAi. Moreover, we examined the cleavage products (miRNAs) generated from synthetic precursors transfected to HeLa cells, as well as the relevant products obtained in reactions with recombinant Dicer *in vitro* (Figure 1). For our study, we have chosen miRNAs heterogeneous in length to analyze possible changes in the pattern of miRNA variants. The results of all analyses were evaluated by high-resolution northern blotting.

### Dicer partners influence endogenous miRNA production

**Figure 2:**
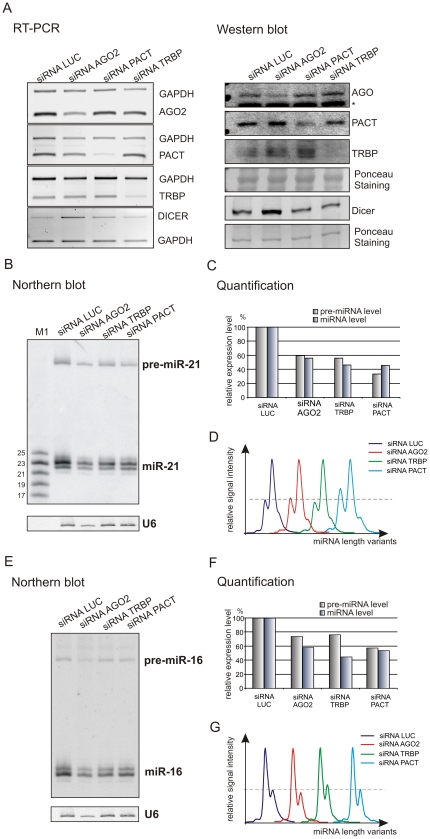
Dicer partners influence endogenous miRNA production. (A) RT-PCR and western blot analyses of the cellular levels of AGO2, PACT, TRBP and Dicer transcripts and proteins 48 h after the second transfection of HeLa cells with LUC, AGO2, PACT and TRBP siRNAs. An asterisk shown in the case of AGO western blot detection indicates cross-reaction with radixin. (B) Northern blotting detection of miR-21 and pre-miR-21 after transfection with specific siRNAs, as indicated in the figure. M1 denotes size marker, end-labeled 17, 19, 21, 23, and 25-nt oligoribonucleotides. Hybridization to U6 RNA provides a loading control. (C) A bar graph showing quantification of the miRNA and pre-miRNA levels detected by northern blotting as presented in B. (D) Quantitative representations of miRNA length variants obtained from the phosphorimaging analyses shown in B. Signals of the highest intensity in each sample were brought to the same height in order to compare contributions from less intense signals. (E–G) Relevant analyses as in B–D, but performed for miR-16 and pre-miR-16.

After the depletion of AGO2, PACT, TRBP with specific siRNA (Figure 2A and Figure S1A), the levels of heterogeneous and highly abundant miR-16 and miR-21 were analyzed. The signal intensities of both miRNAs and pre-miRNAs were substantially decreased compared to controls (Figure 2B, Figure 2C, Figure 2E and Figure 2F). The effect on pre-miRNA levels has not been previously reported or discussed (Table S1). On the contrary, the depletion of Dicer by RNAi in the same experimental system led to an accumulation of both pre-miR-16 and pre-miR-21 (Figure S2). After the depletion of either Dicer partner with specific siRNAs some changes in the level of Dicer protein were observed (Figure 2A). Specifically, the level of Dicer protein decreased considerably after the depletion of PACT and TRBP, while it was increased after depletion of AGO2.

Because we were able to detect miRNAs with single-nucleotide resolution [20], [21], we evaluated miRNA length heterogeneity (Figure 2D and Figure 2G), which primarily reflects the relaxed cleavage specificities of Drosha and Dicer [14], [22]. End-modifications which may contribute to miRNA length heterogeneity to a lesser extent [23], [24] were not taken into consideration in our study. We observed minor differences in miRNA patterns in the cells transfected with siRNA targeting PACT, but only in the case of miR-21 (Figure 2D), and not miR-16. These data suggest that the depletion of Dicer's protein partners influences the production of miRNAs but has only a minor effect on the specificity of Dicer cleavage.

### Dicer partners influence exogenous miRNA production

**Figure 3:**
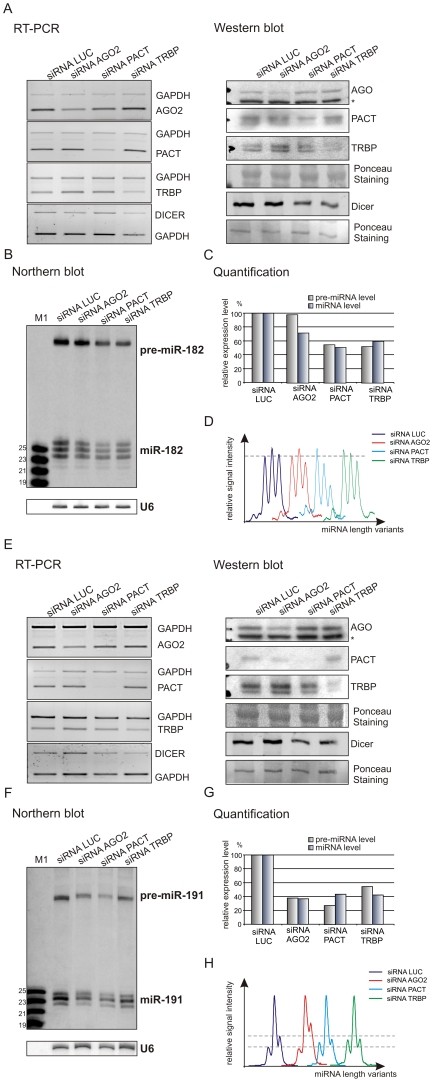
Dicer partners influence exogenous miRNA production. (A) RT-PCR and western blot analyses of cellular levels of AGO2, PACT, TRBP and Dicer transcripts and proteins 72 h after transfection of HeLa cells with LUC, AGO2, PACT and TRBP siRNAs. An asterisk shown in the case of AGO western blot detection indicates cross-reaction with radixin. (B) Northern blotting detection of miR-182 and pre-miR-182 after transfection with specific siRNAs, as indicated in the figure. M1 denotes end-labeled 19, 21, 23, and 25-nt size marker oligoribonucleotides. Hybridization to U6 RNA provides a loading control. (C) Bar graphs showing quantification of the miRNA and pre-miRNA levels detected by northern blotting, as presented in B. (D) Quantitative representations of the miRNA length variants obtained from phosphorimaging analyses from B. Signals of the highest intensity in each sample were brought to the same height in order to compare contributions from less intense signals. (E–H) Relevant analyses as in A–D, but performed for miR-191 and pre-miR-191.

Most miRNAs are stable in cells, and the changes in their steady-state levels may be difficult to detect [12], [25], [26]. To rule out the possibility that the effect of Dicer protein partner depletion on miRNA levels is somehow affected by the high stability of endogenous miRNAs, we used the system for miRNA overexpression and analyzed exogenous miR-182 and miR-191. Again, we chose miRNAs highly heterogeneous in length, but such whose endogenous levels in HeLa cells were hard to detect by northern blotting [14]. The suppression of AGO2, PACT, and TRBP with specific siRNAs (Figure 3A, Figure 3E, Figure S1B, Figure S1C) was followed by cell transfection with miRNA-encoding plasmids (see Materials and Methods). To control for unspecific effects caused by RNAi, transfections with siRNA LUC targeting an irrelevant sequence (luciferase) were also performed. Two independent experiments, for miR-182 and miR-191, showed that the levels of these miRNAs were substantially decreased upon the depletion of Dicer protein partners, and the levels of pre-miRNAs also declined (Figure 3B, Figure 3F). The reduction in miRNA and pre-miRNA levels was similar for both analyzed miRNAs in this experimental system (Figure 3C, Figure 3G). The only exception was the pre-miR-182 expressed in the cells transfected with siRNA AGO2, the level of which did not decrease (Figure 3C). This may result from the insufficient knockdown of AGO2, the remaining amount of which is probably sufficient to form a considerable fraction of an active Dicer complex. The strongest reduction of the miR-191 level was observed in the case of AGO2 suppression and of pre-miR-191 in the case of PACT suppression. Only a minor effect of Dicer protein partner depletion on pre-miRNA cleavage specificity was observed in the case of AGO2 and TRBP knockdown (Figure 3D and Figure 3H), and a substantial change in the miR-182 heterogeneity profile was detected in cells deprived of PACT (Figure 3D). Overall, the results obtained for exogenous miRNAs confirmed those for endogenous miRNAs and showed that the inhibition of Dicer protein partners considerably affects both miRNA and pre-miRNA levels in cells.

### Processing of exogenous pre-miRNAs in cells

In the next step of our study, we wanted to find out whether cellular Dicer with associated proteins would generate the same or different pre-miRNA cleavage patterns as recombinant Dicer acting alone. We addressed this issue by comparing cleavages generated in the same miRNA precursors by cellular Dicer acting within the RLC complex and cleavages generated by recombinant Dicer.

**Figure 4:**
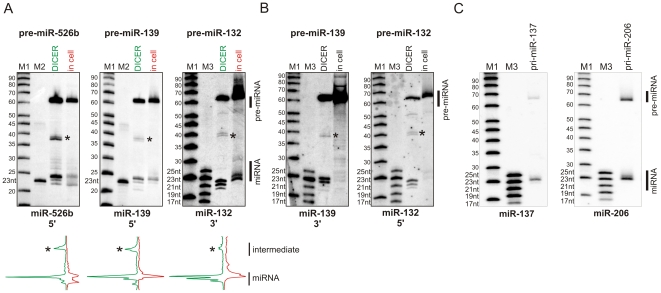
Processing of exogenous pre-miRNA in cells. (A) Northern blot analysis of RNA from *in vitro* reaction (line ‘DICER’) and cellular RNA (line in ‘cell’) with probes specific for miRNA derived from the 5′-arm of pre-miR-526b and pre-miR-139 and the 3′-arm of pre-miR-132. A quantitative representation of the cleavage patterns generated in synthetic pre-miRNAs by recombinant Dicer (green) and cellular Dicer (red) is shown below as peaks obtained from the phosphorimaging analysis. The asterisk marks the ∼40-nt intermediate product of one RNase III Dicer domain cleavage. The black bar on the right side marks the miRNA and pre-miRNA fractions; (B) The same as in A, but antisense probes for miR-139 (3′-arm) and miR-132 (5′-arm) were used; (C) Northern blotting analysis with probes detecting miRNA derived from the 3′-arms of precursors after transfection of HeLa cells with vectors encoding pri-miR-137 and pri-miR-206. The bars on the right side mark the miRNA and pre-miRNA fractions. M1 denotes the low molecular weight RNA marker (USB), M2 – end-labeled 23-nt oligoribonucleotide, and M3 – end-labeled 17, 19, 21, 23, 25-nt oligoribonucleotides.

Synthetic miRNA precursors – pre-miR-132, pre-miR-139 and pre-miR-526b (Table S2) – were transfected to HeLa cells or were treated with recombinant Dicer in a suitable buffer (Figure 1). The products of Dicer cleavages from both experimental systems were analyzed by high-resolution northern blotting, allowing the evaluation of miRNA length variants (Figure 4 and Figure S3). Using the pre-miRNA 5′-arm specific probes, we could detect the miRNAs that were generated by the RNase IIIb domain of Dicer from pre-miR-526b and pre-miR-139, and using the 3′-arm specific probe, we detected miRNAs generated by the RNase IIIa domain of Dicer from pre-miR-132 (Figure 4A). In both systems, Dicer excised miRNAs were heterogeneous in length. The quantitative analysis of cleavage products generated by recombinant and endogenous Dicer showed that among the released products were fragments that corresponded to each other in length; however, the overall Dicer cleavage pattern differed for both experimental systems. The cleavage products generated by endogenous Dicer were barely detectable using miRNA*-specific probes (Figure 4B). The miRNAs* were not detected most likely because they were not selected to be the guide strands and underwent AGO-mediated cleavage followed by degradation [27]–[29]. For the few other pre-miRNAs transfected to Hela cells (e.g., pre-miR-136) no Dicer specific cleavage products were detected using either miRNA or miRNA*-specific probes (Figure S4).

In our previous study in which we analyzed the recombinant Dicer activity on 5′ end-labeled pre-miRNAs we identified ∼40 nt intermediate products [14]. These products, which consisted of one hairpin arm and a terminal loop, resulted from pre-miRNA cleavage by only one RNase III domain of Dicer. Such intermediate products were also observed by other authors [2], [18], [19]. In the present study, the intermediate products were detected using northern blotting technique and are marked by an asterisk in Figure 4A and Figure 4B. Notably, these intermediates were observed only when pre-miRNAs were cleaved by recombinant Dicer and were not observed in the case of synthetic precursors transfected to cells. The ∼40 nt intermediate product was also not detected in cells transfected with vectors expressing pri-miR-137 and pri-miR-206 (Figure 4C) and in the case of the other 14 vector-encoded pri-miRNAs studied earlier [14].

## Discussion

In this study, we investigated the role of Dicer protein partners in the processing of miRNA precursors. We used RNAi to silence individual Dicer protein partners and analyzed the effects of their inhibition on the endogenous and exogenous miRNA production by high-resolution northern blotting.

It has been well documented in the literature that Dicer depletion causes the accumulation of pre-miRNAs and the decrease in the amount of miRNAs (e.g. [7], [30]–[32]). It has also been shown that the level of several pre-miRNAs was not affected by Dicer depletion [33]. In our experimental system, after the depletion of Dicer by RNAi, we observed reduced levels of mature miRNAs and elevated levels of their precursors. Therefore, our observation is in agreement with the majority of previous reports (Table S1). We also detected the decrease in miRNA amount upon AGO2, TRBP, and PACT depletion. Similar results were reported in the literature and explained by the reduced efficiency of dicing caused by a deficiency of Dicer protein partners [5]–[7], [9]. However, upon AGO2, TRBP and PACT depletion we also observed the decrease in the cellular levels of miRNA precursors, which has not been reported so far. As the deficiency of protein partners of Dicer influences the level of Dicer itself (Figure 2A, Figure 3A and Figure 3E), we hypothesize that the pre-miRNA decrease may result from the decreased cellular levels of the functional RLC and a rapid degradation of pre-miRNAs that did not enter the processing complex. The fact that the reduction of TRBP protein level reduces also the level of Dicer protein is in good accordance with the results of others (Table S1), suggesting that the lack of TRBP decreases Dicer stability and/or affects the assembly of the Dicer complex [5], [10]. Similarly, upon PACT depletion the fall in the Dicer level was observed [7]. It is not surprising that the knockdown of TRBP and PACT causes similar effects, as these proteins can interact with Dicer in a similar manner and can act redundantly to some extent [8]. As far as AGO2 depletion is concerned, the observed decrease in pre-miRNAs' level is a novel finding of this study. Earlier, it has been shown that AGO2, which also has a PAZ domain, can bind and cleave pre-miRNAs [34], [35]. It was also proposed that AGO2 may facilitate the access of pre-miRNAs to Dicer [32], [36]. Therefore, the AGO2 binding may contribute to the protection of pre-miRNA from unspecific degradation.

We have performed the siRNA-mediated knockdown of Dicer protein partners and not their knockout, therefore some portion of these proteins remained in the cells. These proteins could form a smaller amount of the fully functional Dicer complex. Therefore, only a portion of pre-miRNAs could be processed and the rest underwent degradation. As a result, we observed smaller amounts of pre-miRNAs and miRNAs.

Our experiments revealed that the silencing of Dicer protein partners had mostly a minor effect on the specificity of Dicer cleavages. The distribution of the length variants of miRNAs differed slightly between the analyzed samples (siRNA targeting AGO2, TRBP and PACT) and the control (siRNA LUC). The changes in the Dicer cleavage pattern could result from the activity of the RNase fraction having changed cleavage specificity when deprived of either protein partner.

The transfection of synthetic pre-miRNAs combined with the northern blotting analysis may be considered another experimental approach to study the Dicer step of miRNA biogenesis. This system, however, needs improvement to make it applicable to a larger number of pre-miRNAs. The synthetic miRNA precursors, used in these experiments, differ in their sequence and structure and may not be equally good substrates for binding and cleavage by the endogenous Dicer complex. It is unlikely that such precursors are fully captured by Dicer. Their substantial portion may be trapped in endosomes. The fraction that escapes endosomes may enter the miRNA biogenesis pathway or may become a target for cellular exo- and endoribonucleases. We do not know what the contribution is of such unspecific processes to the miRNA patterns observed in Figure 4A. We propose, however, that this contribution is not very large because most of the observed northern blot signals belonged to undegraded precursors and miRNA sized products. Recent research shows that siRNAs transfected to cells are primarily intercepted by Dicer, which recognizes 2nt 3′-overhangs, but the efficiency of capture of different siRNAs is highly variable and dependent on the nature of the 3′ overhang sequence [37]. It is also likely that synthetic miRNA precursors transfected to cells may be primarily captured by Dicer that is present in the RLC complex, but the affinity of the Dicer complex to different pre-miRNAs may vary greatly.

The mechanism of Dicer cleavage implies that on the way to the final miRNA-miRNA* duplex resulting from cleavages in both precursor arms by two RNase III domains, the intermediate product may be generated resulting from a more rapid cleavage in one precursor arm by one of the RNase III domains of Dicer. The single-nick intermediate is observed in various amounts for various substrates when synthetic precursors are cleaved with recombinant Dicer [14]. In contrast, in HeLa cells transfected with synthetic pre-miRNAs or miRNA-encoding plasmids, the intermediate product was not observed, suggesting the potential role of protein partners in the synchronization of cleavages generated by individual RNase III domains of Dicer. We considered two alternatives to explain the easy detection of the single-nick intermediates in reactions with recombinant Dicer and the inability to detect this cleavage intermediate in cellular systems. One possibility is that the endogenous Dicer and its protein partners may release the single-nicked precursor to the cytosol, where it undergoes rapid degradation by ribonucleases and escapes detection. The possibility which we consider more likely is that the cleavages generated by two RNase III domains of Dicer are much better synchronized when a precursor undergoes cleavage within RLC than when it is cleaved by recombinant Dicer alone.

## Materials and Methods

### Oligonucleotides

The pre-miRNAs used for transfection, pre-miR-132, pre-miR-136, pre-miR-139 and pre-miR-526b, were chemically synthesized (Curevac) and purified by polyacrylamide gel electrophoresis. Their sequences, together with sequences of oligoribonucleotides used for siRNA preparation (Metabion) and oligodeoxynucleotides used as probes for northern blotting (IBB Warsaw), are presented in supplementary tables (Table S2, Table S3 and Table S4).

### RNA cleavage assay using recombinant Dicer

Prior to reaction with recombinant human Dicer (Ambion), ∼4 pmols of unlabeled RNAs (pre-miRNAs) was incubated with Dicer (1 U) at 37°C for 60 min. The reactions were stopped by adding an equal volume of gel loading buffer (7.5 M urea and 20 mM EDTA with dyes), and aliquots were subjected to northern blot analysis.

### Cell culture

HeLa cells were obtained from the American Type Culture Collection (ATCC) and grown in Dulbecco's Modified Eagle's Medium (Lonza) supplemented with 10% fetal bovine serum (Sigma) and antibiotic–antimycotic solution (Sigma) at 37°C in a humidified atmosphere of 5% CO_2_.

### DNA, siRNA and pre-miRNA transfection

HeLa cells were transfected using Lipofectamine 2000 (Invitrogen) according to the manufacturer's recommendations for DNA and siRNA transfections. For miRNA overexpression, the cells were grown to 90% confluence, transfected with plasmid constructs (1 µg/ml) expressing miRNA precursors (pri-miR-182 (Open Biosystems), pri-miR-191, pri-miR-137 or pri-miR-206 (System Biosciences)), and harvested 24 hours after transfection. The plasmid constructs (Open Biosystems, System Biosciences) contain pri-miRNA sequences in their natural genome context to ensure biologically relevant interactions with the endogenous processing machinery. For the depletion of AGO2, TRBP, PACT and Dicer by RNAi, specific siRNAs (Table S3) were used. As a control, the siRNA targeting a non-relevant sequence was also used where indicated (luciferase sequence, LUC). In brief, sense and antisense siRNA strands (Metabion) were annealed and appropriate siRNA duplexes were used to transfect ∼50% confluent HeLa cells at a final concentration of 50 nM. Two independent siRNA transfections were performed with the same concentration of specific siRNAs but with different timing in two experimental approaches. For the analysis of endogenous miRNAs, the second siRNA transfection was repeated the following day and RNAs and proteins were isolated 48 h after the second transfection. For the exogenous miRNAs (miRNA overexpression system), the cell cultures were split and re-plated 24 h after the first transfection and re-transfected with siRNAs 24 h later (the second transfection was done 48 h after the first transfection). Moreover, 48 h after the second transfection, the HeLa cells were transfected with plasmid constructs encoding miRNA precursors, pre-miR-191 (System Biosciences) and pre-miR-182 (Open Biosystems), as described above for miRNA overexpression. RNA and protein analyses were done 72 h after the second transfection with specific siRNAs.

The transfection of synthetic miRNA precursors (Table S2) was performed using Oligofectamine (Invitrogen) according to the manufacturer's instructions. Phosphorylated RNAs were used at a final concentration of 3.5 nM. The RNAs isolated after all of the transfections were analyzed by northern blotting.

### RNA isolation and RT-PCR

Total RNA from HeLa cells was extracted using TRI Reagent (MRC, Inc., BioShop) according to the protocol provided by the manufacturer. To examine the silencing activity of siRNA, RNAs extracted from appropriate samples (1 µg) were subjected to cDNA synthesis with random primers (Promega) using Super Script II Reverse Transcriptase (Invitrogen). We have designed primers for RT-PCR to amplify AGO2 products having 165 bp, TRBP products having 235 bp, PACT products having 237 bp and Dicer products having 537 bp.

The primer sequences and conditions for the PCR amplification of AGO2, PACT, TRBP and Dicer were the following: AGO2_F 5′-CAGTGCGTGCAGATGAAGAA and AGO2_R 5′-ATGGACGTCTGCTCCCAGAA, 94–60–72°C, 15 sec, 25 cycles; PACT_F 5′-ACGAATACGGCATGAAGACC and PACT_R 5′-TGGAAGGGTCAGGCATTAAG, 94-55-72°C, 15 sec, 25 cycles; TRBP_F 5′-GCACCTGGGATTCTCTACGA and TRBP_R 5′-GCAGAGCCATGACACACAGT, 94-55-72°C, 15 sec, 25 cycles; Dicer_F 5′-TTAACCAGCTGTGGGGAGAGGGCTG and Dicer_R 5′-AGCCAGCGATGCAAAGATGGTGTTG, 94-61-72°C, 15 sec, 22 cycles. PCR amplification for GAPDH normalization was performed using GAPDH_F 5′-TCCACCACCCTGTTGCTCTA and GAPDH_R 5′-ACCACAGTCCATGCCATCAC primers [38] in multiplex reactions together with either AGO2, TRBP or PACT primers. For Dicer, for normalization the following primers were used: GAPDH_Fd 5′ GAAGGTGAAGGTCGGAGTC and GAPDH_Rd 5′-GAAGATGGTGATGGGATTTC.

### Northern blot analysis

High-resolution northern blotting was performed as previously described [14], [20], [21]. Briefly, total RNA extracted from HeLa cells (20–30 µg) or RNA aliquots (∼40 fmole) obtained in Dicer cleavage reactions *in vitro* were resolved on a denaturing polyacrylamide gel (12% PAA) in 0.5x TBE. The RNAs were transferred to a GeneScreen Plus hybridization membrane (PerkinElmer) using semi-dry electroblotting (Sigma-Aldrich), immobilized with UV irradiation (120 mJ/cm^2^) (UVP) and then baked in an oven at 80°C for 30 min. The membranes were probed with specific DNA oligonucleotides (Table S4). The oligonucleotides were labeled with [γ^32^P] ATP (5000 Ci/mmol; Hartmann Analytics) using OptiKinase (USB). Hybrydizations were carried out at 37°C overnight in a buffer containing 5x SSC, 1% SDS and 1x Denhardt's solution. The marker lanes contained a mixture of radiolabeled RNA oligonucleotides (ORNs: 17-, 19-, 21-, 23- and 25-nt) and/or RNA low molecular weight marker (USB Corporation). Hybridizations to U6 RNA provided loading controls. Specifically, U6 snRNA was hybridized to the same blots as all miRNA probes and hybridization intensities from miRNA and pre-miRNA detections were normalized to the U6 RNA signal. The relative levels of miRNA and pre-miRNA in RNA samples extracted from cells transfected with siRNA targeting a non-relevant sequence (luciferase sequence, LUC) were used as our controls. The results are presented in % and compared to the control having 100%. Radioactive signals were quantified by phosphorimaging (Multi Gauge v3.0; Fujifilm). The relative change in the contribution from individual miRNA length variants between samples was considered to be minor when individual northern blotting signal intensities in the sample lane differed from the intensity of the corresponding signals in the control lane by ±5–10%, while a difference of above ±10% was described as a substantial change.

### Western blotting

After the second transfection with specific siRNAs, the HeLa cells were lysed with 1X PBS buffer supplemented with protease inhibitor cocktail (Roche); 48 h and 72 h after the second transfection of HeLa cells with specific siRNAs for the analysis of endogenous and exogenous miRNAs, respectively. For AGO, PACT, TRBP detections a total of 100 µg of protein was diluted in sample buffer containing 2-mercaptoethanol and boiled for 5 min. For the detection of Dicer, a total of 50 µg of protein was prepared in the same way. Protein lysates were separated by 10% SDS-PAGE electrophoresis with the exception of the electrophoresis for Dicer detection. The latter was done in 4% Tris–acetate SDS-PAGE and run in XT Tricine buffer (Bio-Rad), as described previously for huntingtin [39]. After electrophoreses all the proteins were electrotransferred onto a nitrocellulose membrane (Sigma). All steps of immunodetection were performed on a SNAPid (Millipore) in buffer containing 0.2% nonfat milk in PBS/0.1% Tween 20, and the membranes were washed in PBS/Tween. For AGO, PACT and TRBP detection, appropriate blots were probed with the primary antibodies anti-human AGO, PACT and TRBP (diluted 1∶300), respectively, and then with HRP-conjugated secondary antibodies (1∶500, Sigma). The antibodies for AGO (2A8) were kindly provided by Z. Mourelatos [40], and the PACT and TRBP antibodies were a gift from N.V. Kim [7]. For Dicer detection the rabbit polyclonal primary antibody was used (1∶1000, Cell Signaling Technology) and the HRP-conjugated secondary antibody (1∶500, Sigma). The immunoreaction was detected using Pierce ECL Western Blotting Substrate (Thermo Scientific). The staining of membrane proteins with Ponceau S (Sigma) provided a loading control.

Western blotting for the detection of Dicer presented as supplementary data (Figure S2) was performed with the following modifications. A total of 40 µg of protein was analyzed 48 h after the second transfection on 12% SDS-PAGE. The primary antibodies anti-human Dicer (1∶300, Abcam) and biotinylated secondary antibodies (1∶500 Sigma) were used; the membranes were incubated with a streptavidin–AP conjugate (1∶2000, Millipore) and immunoreactive bands were visualized using the Sigma Fast BCIP/NBT kit (Sigma). GAPDH detected by anti-GAPDH antibodies (1∶5000, Millipore) provided a loading control.

## Supporting Information

Figure S1 **RT-PCR quantitative analyses of AGO2, PACT and TRBP transcript levels.** (A) Cellular levels of AGO2, PACT and TRBP transcripts 48 h after second transfection of HeLa cells with LUC, AGO2, PACT and TRBP siRNAs evaluated for endogeneous miRNA analysis. (B and C) Cellular levels of AGO2, PACT and TRBP transcripts 72 h after second transfection of HeLa cells with LUC, AGO2, PACT and TRBP siRNAs evaluated for exogeneous miRNA analysis (for pri-miR-182 (B); for pri-miR-191(C)). Error bars represent standard deviation. The data gather results from two independent experiments.
(TIF)

Figure S2 **The influence of Dicer depletion on pre-miRNA and miRNA levels.** High-resolution northern blot analysis of the endogeneous pre-miR-16 and pre-miR-21 (A) and miR-16 and miR-21 (B) in HeLa cells after depletion of Dicer by RNAi, as indicated in the figures. M1 denotes low molecular weight RNA marker (USB). M2 denotes size marker, end-labeled 19, 21, 23, 25-nt oligoribonucleotides. Hybridization to U6 RNA and EtBr staining provide loading controls. Western blot analysis depicting Dicer protein levels, endogenous and decreased in HeLa cells after depletion by siRNA, is also shown. GAPDH protein level provides a loading control.
(TIF)

Figure S3 **A scheme depicting predicted structures of the miRNA precursors used in this study.** (A) Fragments of pri-miRNA structures used to analyze Dicer cleavage specificity with indicated regions where the specific radioactive probe hybridized. (B) Pre-miRNA sequences used in transfection experiments, their predicted folding and regions where the specific radioactive probe hybridized (green – 5′ arm specific probe, blue – 3′ arm specific probe).
(TIF)

Figure S4 **Analysis of endogenous Dicer activity in Hela cells on pre-miR-136.** Northern blot analysis of the products generated from synthetic pre-miR-136 that was transfected into HeLa cells using Oligofectamine. RNA was isolated 24 h after transfection with the indicated pre-miRNA and northern blotted (lanes labeled “in cell”) with a specific probe for miRNA derived from 5′-arm or 3′-arm of pre-miR-136, as indicated by the label 5′ or 3′. The asterisk marks the ∼40-nt intermediate product of one RNase III Dicer domain cleavage. The black bar on the right side marks the miRNA and pre-miRNA fractions. For comparison, the products of the reactions that contained unlabeled pre-miRNA with recombinant Dicer were also analyzed by northern blotting (lanes labeled DICER). M1 denotes low molecular weight RNA marker (USB), M2 – size marker, end-labeled 17, 19, 21, 23, 25-nt oligoribonucleotides.
(TIF)

Table S1 **Similarities and discrepancies between selected published results reporting the effects of depletion of Dicer and its protein partners on the level of AGO2, PACT, TRBP, DICER, miRNA and pre-miRNA.** Denotation: Arrows indicate either increase or decrease in the protein level of the AGO2, PACT, TRBP, DICER, and the appropriate change in miRNA and pre-miRNA levels. An equal sign indicates no change. Shadowed cells present the expected effect of targeting the particular gene with specific siRNAs.
(PDF)

Table S2 **The sequences of synthetic pre-miRNAs.**
(PDF)

Table S3 **The sequences of RNA oligonucleotides used for siRNA preparation.**
(PDF)

Table S4 **Oligodeoxynucleotide sequences used as northern blot probes.** The sequences of the miRNA specific probes were designed to be fully complementary to miRNA or miRNA* sequence based on data deposited in miRBase (Griffiths-Jones et al. 2006).
(PDF)
