# Supplementary figures and images for: The Role of Dicer Protein Partners in the Processing of MicroRNA Precursors
Source: PLoS One. 2011 Dec 6;6(12):e28548. doi: 10.1371/journal.pone.0028548 (PMC3232248; doi:10.1371/journal.pone.0028548)

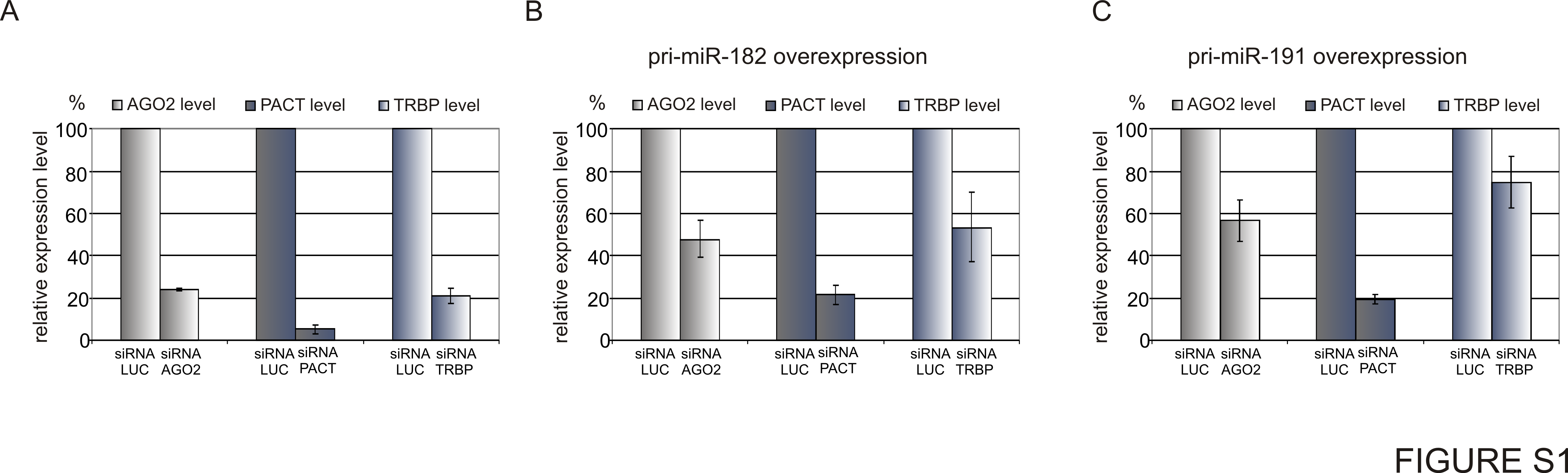

Supplement: Figure S1 — RT-PCR quantitative analyses of AGO2, PACT and TRBP transcript levels. (A) Cellular levels of AGO2, PACT and TRBP transcripts 48 h after second transfection of HeLa cells with LUC, AGO2, PACT and TRBP siRNAs evaluated for endogeneous miRNA analysis. (B and C) Cellular levels of AGO2, PACT and TRBP transcripts 72 h after second transfection of HeLa cells with LUC, AGO2, PACT and TRBP siRNAs evaluated for exogeneous miRNA analysis (for pri-miR-182 (B); for pri-miR-191(C)). Error bars represent standard deviation. The data gather results from two independent experiments. (TIF) [file pone.0028548.s001.tif]

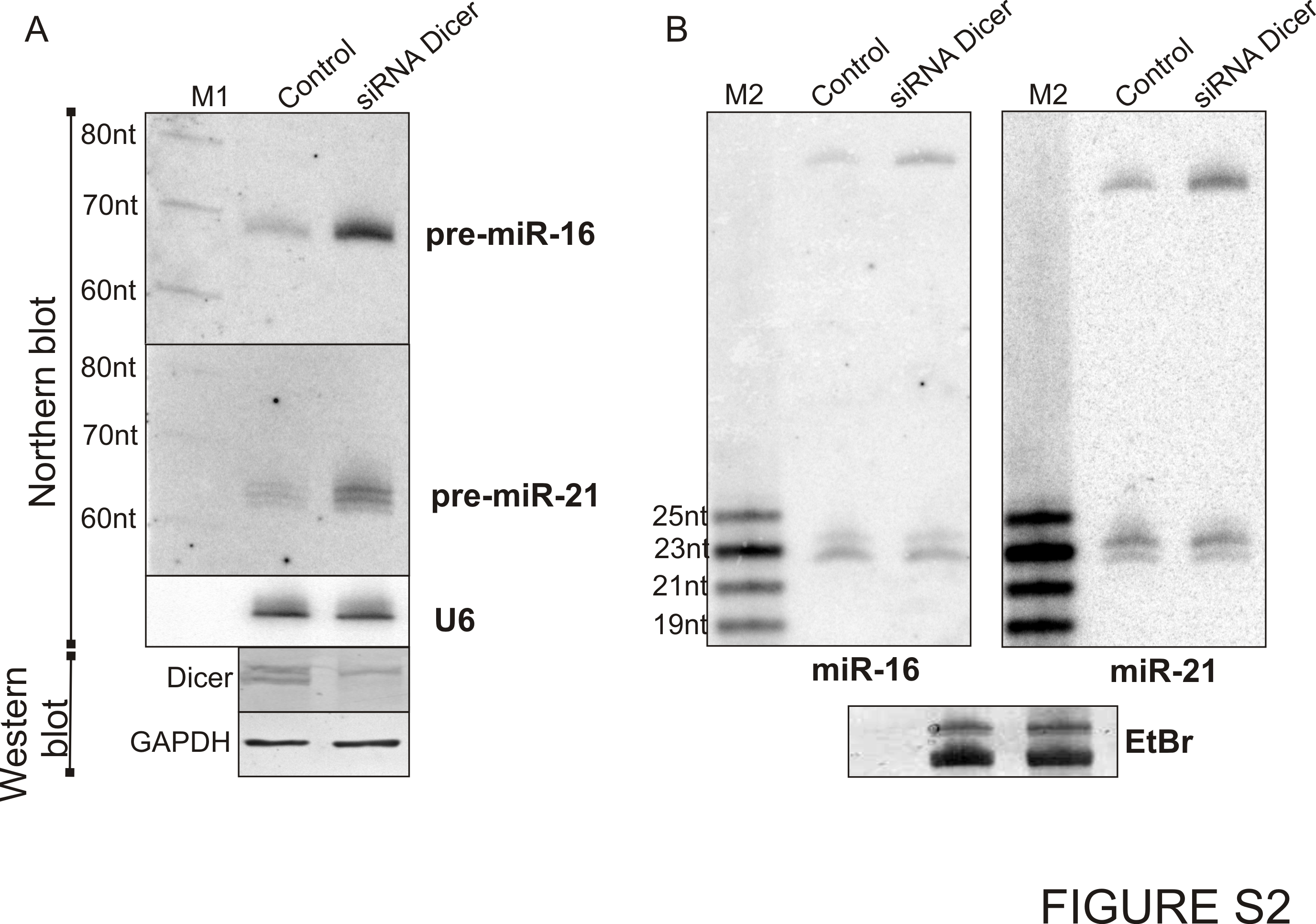

Supplement: Figure S2 — The influence of Dicer depletion on pre-miRNA and miRNA levels. High-resolution northern blot analysis of the endogeneous pre-miR-16 and pre-miR-21 (A) and miR-16 and miR-21 (B) in HeLa cells after depletion of Dicer by RNAi, as indicated in the figures. M1 denotes low molecular weight RNA marker (USB). M2 denotes size marker, end-labeled 19, 21, 23, 25-nt oligoribonucleotides. Hybridization to U6 RNA and EtBr staining provide loading controls. Western blot analysis depicting Dicer protein levels, endogenous and decreased in HeLa cells after depletion by siRNA, is also shown. GAPDH protein level provides a loading control. (TIF) [file pone.0028548.s002.tif]

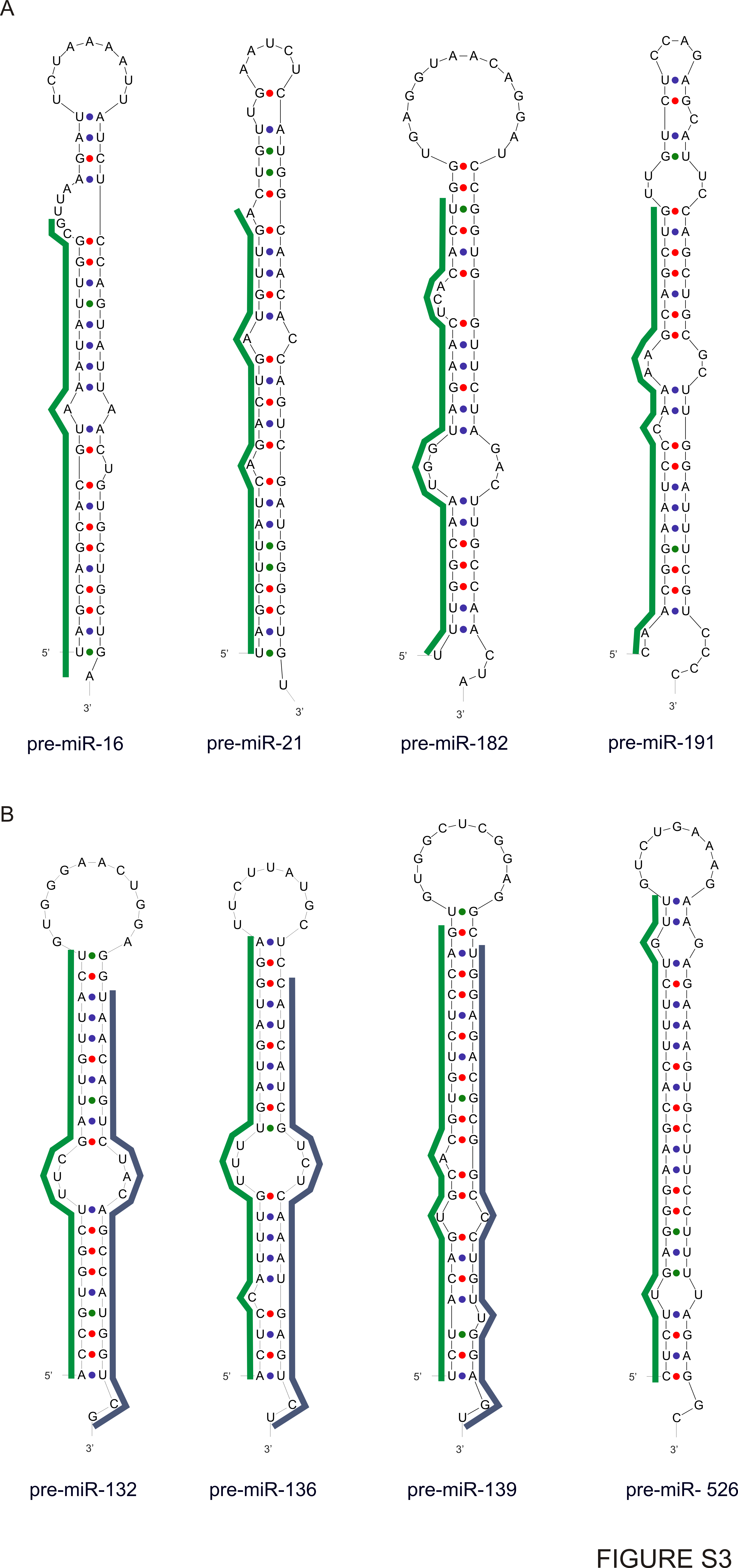

Supplement: Figure S3 — A scheme depicting predicted structures of the miRNA precursors used in this study. (A) Fragments of pri-miRNA structures used to analyze Dicer cleavage specificity with indicated regions where the specific radioactive probe hybridized. (B) Pre-miRNA sequences used in transfection experiments, their predicted folding and regions where the specific radioactive probe hybridized (green – 5′ arm specific probe, blue – 3′ arm specific probe). (TIF) [file pone.0028548.s003.tif]

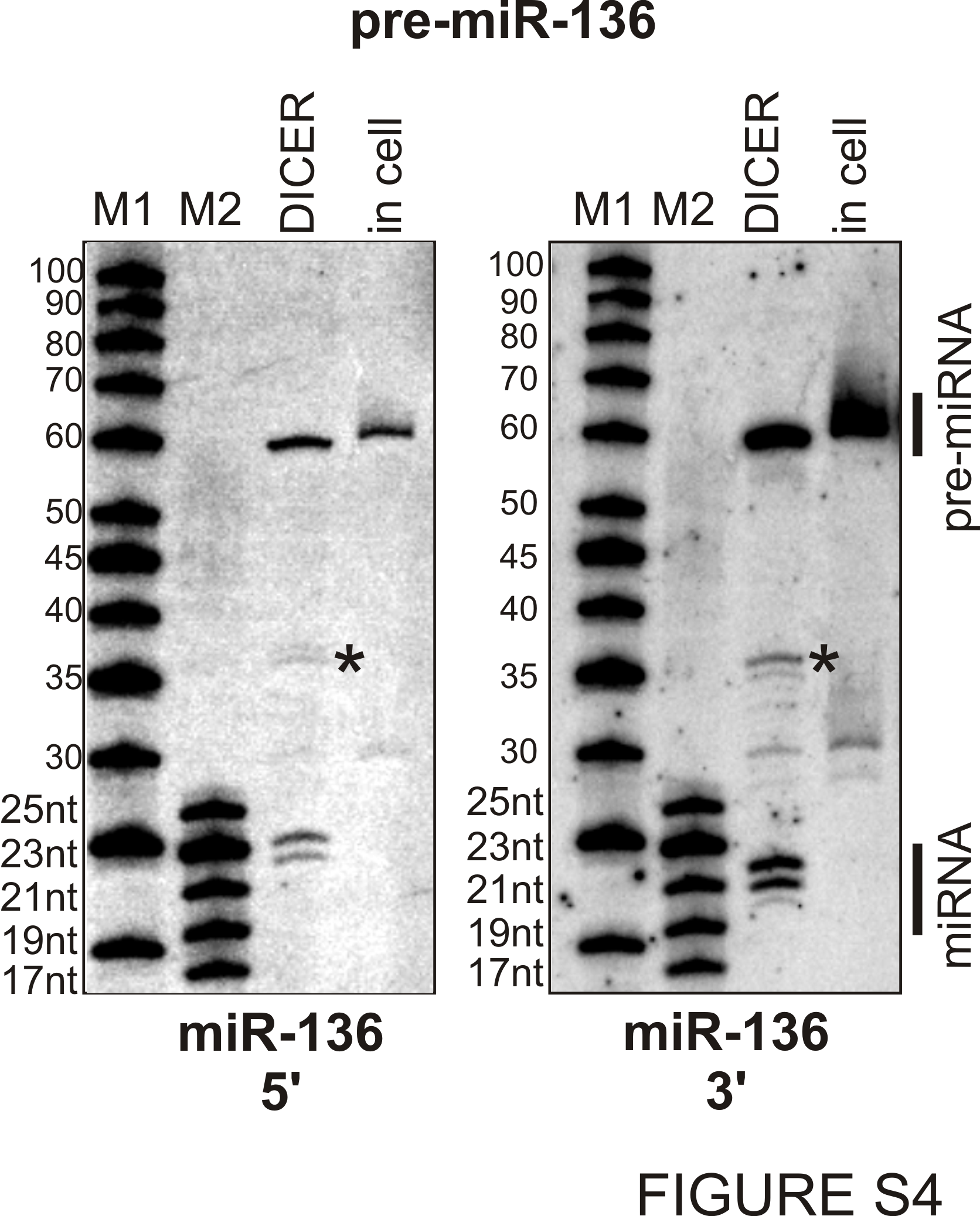

Supplement: Figure S4 — Analysis of endogenous Dicer activity in Hela cells on pre-miR-136. Northern blot analysis of the products generated from synthetic pre-miR-136 that was transfected into HeLa cells using Oligofectamine. RNA was isolated 24 h after transfection with the indicated pre-miRNA and northern blotted (lanes labeled “in cell”) with a specific probe for miRNA derived from 5′-arm or 3′-arm of pre-miR-136, as indicated by the label 5′ or 3′. The asterisk marks the ∼40-nt intermediate product of one RNase III Dicer domain cleavage. The black bar on the right side marks the miRNA and pre-miRNA fractions. For comparison, the products of the reactions that contained unlabeled pre-miRNA with recombinant Dicer were also analyzed by northern blotting (lanes labeled DICER). M1 denotes low molecular weight RNA marker (USB), M2 – size marker, end-labeled 17, 19, 21, 23, 25-nt oligoribonucleotides. (TIF) [file pone.0028548.s004.tif]
