# Supplementary material for: The Role of Dicer Protein Partners in the Processing of MicroRNA Precursors
Source: PLoS One. 2011 Dec 6;6(12):e28548. doi: 10.1371/journal.pone.0028548 (PMC3232248; doi:10.1371/journal.pone.0028548)
Supplement: Table S1 — Similarities and discrepancies between selected published results reporting the effects of depletion of Dicer and its protein partners on the level of AGO2, PACT, TRBP, DICER, miRNA and pre-miRNA. Denotation: Arrows indicate either increase or decrease in the protein level of the AGO2, PACT, TRBP, DICER, and the appropriate change in miRNA and pre-miRNA levels. An equal sign indicates no change. Shadowed cells present the expected effect of targeting the particular gene with specific siRNAs. (PDF) [file pone.0028548.s005.pdf]

Table S1. Similarities and discrepancies between selected published results reporting the effects of depletion of Dicer and its protein partners on the level of AGO2, PACT, TRBP, DICER, miRNA and pre-miRNA.

Denotation: Arrows indicate either increase or decrease in the protein level of the AGO2, PACT, TRBP, DICER, and the appropriate change in miRNA and pre-miRNA levels. An equal sign indicates no change. Shadowed cells present the expected effect of targeting the particular gene with specific siRNAs.

	AGO2 level	PACT level	TRBP level	DICER level	miRNA level	pre-miRNA level
**AGO2 deficiency**	↓	↑ = in agreement with the results of (Lee *et al.* 2006) but not commented by the authors	↑ in agreement with the results of (Lee *et al.* 2006)	↑ = in agreement with the results of (Lee *et al.* 2006; Vickers *et al.* 2007)	↓ in agreement with the results of (O'Carroll *et al.* 2007)	↓ in contrast to the results of (O'Carroll *et al.* 2007)
**PACT deficiency**	=	↓	↓ = in contrast to the results of (Lee *et al.* 2006)	↓ in agreement with the results of (Lee *et al.* 2006)	↓ in agreement with the results of (Lee *et al.* 2006)	↓ in agreement with the results of (Lee *et al.* 2006) but not discussed by the authors
**TRBP deficiency**	= in agreement with the results of (Chendrimada *et al.* 2005)	= in agreement with the results of (Lee *et al.* 2006)	↓	↓ in agreement with the results of (Chendrimada *et al.* 2005; Melo *et al.* 2009)	↓ in agreement with the results of (Chendrimada *et al.* 2005; Lee *et al.* 2006; Melo *et al.* 2009)	↓
**DICER deficiency**	= in agreement with the results of (Chendrimada *et al.* 2005)	= in agreement with the results of (Lee *et al.* 2006)	= in agreement with the results of (Lee *et al.* 2006)	↓	↓ in agreement with the results of (Landthaler *et al.* 2004; Monticelli *et al.* 2005; Cummins *et al.* 2006; Lee *et al.* 2006; O'Carroll *et al.* 2007)	↑

REFERENCES

Chendrimada, T. P., Gregory, R. I., Kumaraswamy, E., Norman, J., Cooch, N., Nishikura, K. and Shiekhattar, R. (2005). "TRBP recruits the Dicer complex to Ago2 for microRNA processing and gene silencing." Nature 436(7051): 740-744.

Cummins, J. M., He, Y., Leary, R. J., Pagliarini, R., Diaz, L. A., Jr., Sjoblom, T., Barad, O., Bentwich, Z., Szafranska, A. E., Labourier, E., Raymond, C. K., Roberts, B. S., Juhl, H., Kinzler, K. W., Vogelstein, B. and Velculescu, V. E. (2006). "The colorectal microRNAome." Proc Natl Acad Sci U S A 103(10): 3687-3692.

Landthaler, M., Yalcin, A. and Tuschl, T. (2004). "The human DiGeorge syndrome critical region gene 8 and Its D. melanogaster homolog are required for miRNA biogenesis." Curr Biol 14(23): 2162-2167.

Lee, Y., Hur, I., Park, S. Y., Kim, Y. K., Suh, M. R. and Kim, V. N. (2006). "The role of PACT in the RNA silencing pathway." EMBO J 25(3): 522-532.

Melo, S. A., Ropero, S., Moutinho, C., Aaltonen, L. A., Yamamoto, H., Calin, G. A., Rossi, S., Fernandez, A. F., Carneiro, F., Oliveira, C., Ferreira, B., Liu, C. G., Villanueva, A., Capella, G., Schwartz, S., Jr., Shiekhattar, R. and Esteller, M. (2009). "A TARBP2 mutation in human cancer impairs microRNA processing and DICER1 function." Nat Genet 41(3): 365-370.

Monticelli, S., Ansel, K. M., Xiao, C., Socci, N. D., Krichevsky, A. M., Thai, T. H., Rajewsky, N., Marks, D. S., Sander, C., Rajewsky, K., Rao, A. and Kosik, K. S. (2005). "MicroRNA profiling of the murine hematopoietic system." Genome Biol 6(8): R71.

O'Carroll, D., Mecklenbrauker, I., Das, P. P., Santana, A., Koenig, U., Enright, A. J., Miska, E. A. and Tarakhovsky, A. (2007). "A Slicer-independent role for Argonaute 2 in hematopoiesis and the microRNA pathway." Genes Dev 21(16): 1999-2004.

Vickers, T. A., Lima, W. F., Nichols, J. G. and Crooke, S. T. (2007). "Reduced levels of Ago2 expression result in increased siRNA competition in mammalian cells." Nucleic Acids Res 35(19): 6598-6610.
