# Supplementary material for: The Role of Dicer Protein Partners in the Processing of MicroRNA Precursors
Source: PLoS One. 2011 Dec 6;6(12):e28548. doi: 10.1371/journal.pone.0028548 (PMC3232248; doi:10.1371/journal.pone.0028548)
Supplement: Table S2 — The sequences of synthetic pre-miRNAs. (PDF) [file pone.0028548.s006.pdf]

Table S2. The sequences of synthetic pre-miRNAs

Name	Sequence (5`→3`)	References
pre-miR-132	ACCGUGGCUUUCGAUUGUUACUGUGGGAACU GGAGGUAACAGUCUACAGCCAUGGUCG	(Starega-Roslan *et al.* 2011)
pre-miR-136	ACUCCAUUUGUUUUGAUGAUGGAUUCUUAUG CUCCAUCAUCGUCUCAAAUGAGUCU	(Starega-Roslan *et al.* 2011)
pre-miR-139	UCUACAGUGCACGUGUCUCCAGUGUGGCUCG GAGGCUGGAGACGCGGCCCUGUUGGAGU	(Starega-Roslan *et al.* 2011)
pre-miR-526b	CUCUUGAGGGAAGCACUUUCUGUUGUCUGAA AGAAGAGAAAGUGCUUCCUUUUAGAGGC	(Starega-Roslan *et al.* 2011)

Starega-Roslan, J., Krol, J., Koscianska, E., Kozlowski, P., Szlachcic, W. J., Sobczak, K. and Krzyzosiak, W. J. (2011). "Structural basis of microRNA length variety." Nucleic Acids Res 39(1): 257-268.
