# Supplementary material for: The Role of Dicer Protein Partners in the Processing of MicroRNA Precursors
Source: PLoS One. 2011 Dec 6;6(12):e28548. doi: 10.1371/journal.pone.0028548 (PMC3232248; doi:10.1371/journal.pone.0028548)
Supplement: Table S3 — The sequences of RNA oligonucleotides used for siRNA preparation. (PDF) [file pone.0028548.s007.pdf]

Table S3. The sequences of RNA oligonucleotides used for siRNA preparation

Name	Sequence (5`→3`)	References
sense TRBP	UUCACCAUGACCUGUCGAGTT	(Chendrimada *et al*. 2005)
antisense TRBP	(p)CUCGACAGGUCAUGGUGAATT	
sense PACT	GAACCAGCUUAAUCCUAUUTT	(Lee *et al*. 2006)
antisense PACT	(p)AAUAGGAUUAAGCUGGUUCTT	
sense AGO2	ACAGAUUCCCAAAGGGUAATT	Our design
antisense AGO2	(p)UAACCCUUUGGGAAUCUGUTT	
sense LUC	CGUACGCGGAAUACUUCGAUU	(Chen *et al*. 2008)
antisense LUC	(p)UCGAAGUAUUCCGCGUACGUU	
sense DICER	GGCACCCAUCUCUAAUUAUUT	(Landthaler *et al*. 2004)
antisense DICER	(p)AUAAUUAGAGAUGGGUGCCUT	

Chen, P. Y., Weinmann, L., Gaidatzis, D., Pei, Y., Zavolan, M., Tuschl, T. and Meister, G. (2008). "Strand-specific 5'-O-methylation of siRNA duplexes controls guide strand selection and targeting specificity." RNA 14(2): 263-274.

Chendrimada, T. P., Gregory, R. I., Kumaraswamy, E., Norman, J., Cooch, N., Nishikura, K. and Shiekhattar, R. (2005). "TRBP recruits the Dicer complex to Ago2 for microRNA processing and gene silencing." Nature 436(7051): 740-744.

Landthaler, M., Yalcin, A. and Tuschl, T. (2004). "The human DiGeorge syndrome critical region gene 8 and Its D. melanogaster homolog are required for miRNA biogenesis." Curr Biol 14(23): 2162-2167.

Lee, Y., Hur, I., Park, S. Y., Kim, Y. K., Suh, M. R. and Kim, V. N. (2006). "The role of PACT in the RNA silencing pathway." EMBO J 25(3): 522-532.
