# Supplementary material for: The Role of Dicer Protein Partners in the Processing of MicroRNA Precursors
Source: PLoS One. 2011 Dec 6;6(12):e28548. doi: 10.1371/journal.pone.0028548 (PMC3232248; doi:10.1371/journal.pone.0028548)
Supplement: Table S4 — Oligodeoxynucleotide sequences used as northern blot probes. The sequences of the miRNA specific probes were designed to be fully complementary to miRNA or miRNA* sequence based on data deposited in miRBase (Griffiths-Jones et al. 2006). (PDF) [file pone.0028548.s008.pdf]

Table S4. Oligodeoxynucleotide sequences used as northern blot probes.

The sequences of the miRNA specific probes were designed to be fully complementary to miRNA or miRNA* sequence based on data deposited in miRBase (Griffiths-Jones *et al.* 2006).

Name	Sequence (5`→3`)
miR-16	CGCCAATATTTACGTGCTGCTAA
miR-21	TCAACATCAGTCTGATAAGCTA
miR-132-5p	AGTAACAATCGAAAGCCACGGT
miR-132-3p	CGACCATGGCTGTAGACTGTTA
miR-136-5p	TCCATCATCAAAACAAATGGAGT
miR-136-3p	AGACTCATTTGAGACGATGATG
miR-137	CTACGCGTATTCTTAAGCAATAA
miR-139-5p	CTGGAGACACGTGCACTGTAGA
miR-139-3p	ACTCCAACAGGGCCGCGTCTCCA
miR-191	CAGCTGCTTTTGGGATTCCGTTG
miR-182	AGTGTGAGTTCTACCATTGCCAAA
miR-206	CCACACACTTCCTTACATTCCA
miR-526b	AACAGAAAGTGCTTCCCTCAAGAG
U6 (loading control)	GCAGGGGCCATGCTAATCTTCTCTGTATCG

Griffiths-Jones, S., Grocock, R. J., van Dongen, S., Bateman, A. and Enright, A. J. (2006). "miRBase: microRNA sequences, targets and gene nomenclature." Nucleic Acids Res 34(Database issue): D140-144.
